# Media Coverage of Medical Journals: Do the Best Articles Make the News?

**DOI:** 10.1371/journal.pone.0085355

**Published:** 2014-01-17

**Authors:** Senthil Selvaraj, Durga S. Borkar, Vinay Prasad

**Affiliations:** 1 Department of Medicine, Brigham and Women's Hospital, Boston, Massachusetts, United States of America; 2 Department of Medicine, Beth Israel Deaconess - Brockton, Brockton, Massachusetts, United States of America; 3 Medical Oncology Branch, National Cancer Institute, National Institutes of Health, Bethesda, Maryland, United States of America; College of Pharmacy, University of Florida, United States of America

## Abstract

**Background:**

News coverage of medical research is followed closely by many Americans and affects the practice of medicine and influence of scientific research. Prior work has examined the quality of media coverage, but no investigation has characterized the choice of stories covered in a controlled manner. We examined whether the media systematically covers stories of weaker study design.

**Methods:**

We compared study characteristics of 75 clinically-oriented journal articles that received coverage in the top five newspapers by circulation against 75 clinically-oriented journal articles that appeared in the top five medical journals by impact factor over a similar timespan. Subgroup analysis was performed to determine whether differences between investigations from both sources varied by study type (randomized controlled trial [RCT] or observational study).

**Results:**

Investigations receiving coverage from newspapers were less likely to be RCTs (17% vs. 35%, p = 0.016) and more likely to be observational studies (75% vs. 47%, p<0.001). No difference was observed in number of people studied (median: 1034 vs. 1901, p = 0.14) or length of follow-up (median: 1.80 vs. 1.00 years, p = 0.22). In subgroup analysis, observational studies from the media used smaller sample sizes (median: 1984 vs. 21136, p = 0.029) and were more likely to be cross-sectional (71% vs. 31%, p<0.001), while no differences were observed for RCTs.

**Conclusions:**

Newspapers were more likely to cover observational studies and less likely to cover RCTs than high impact journals. Additionally, when the media does cover observational studies, they select articles of inferior quality. Newspapers preferentially cover medical research with weaker methodology.

## Introduction

Reporting on a large observational study noting a link between statin use and decreased cancer mortality [1], one headline read, “Statins Cut Mortality in Cancer Patients.” [2] Another major news agency proclaimed, “Statin-takers Less Likely to Die from Cancer.” [3] An article in the LA Times entitled, “Statins may lower risk of cancer death,” quotes the study's lead author saying, “Regular statin use before and after a diagnosis of cancer could theoretically reduce cancer-related mortality.” [4] *Health News Review*, an independent organization charged with critically evaluating health care journalism, concluded that most of the news coverage on this article had been “botched,” [5] encouraging readers to draw dubious causal conclusions from observational data.

The coverage of this study on a potential cancer therapy came at the expense of other studies. In the same issue of the *New England Journal of Medicine* in which this study appeared, a large randomized controlled trial (RCT) found that Trastuzumab emtansine (T-DM1) provided an overall mortality benefit over standard of care among women with advanced, HER2-positive breast cancer, which had progressed on first line therapy [6]. While T-DM1 did receive news coverage, the number of stories on this topic were far fewer than the investigation of statins (77 and 311, respectively, per Google News Search on April 18, 2013). Many of the news agencies that reported the association between statins and cancer death did not cover the link between T-DM1 and reduced cancer death. Either article could have received news coverage, but the observational study preferentially did.

The validity of health care journalism is a product of both the quality of the coverage [7], [8] as well as the choice of stories covered. Independent organizations, such as *Health News Review*, critically assess the former; however, to our knowledge, no investigation has documented the latter in a controlled fashion. Here, we empirically compare research articles covered by highly circulated newspapers to original articles appearing in high impact journals over the same time period. Specifically, we asked whether news outlets systematically cover stories of weaker methodological quality, preferring observational studies to RCTs.

## Methods

We constructed a database of 75 original medical articles recently covered by widely circulated newspapers and a corresponding set of 75 original medical articles recently published in high impact journals over the same time period. In doing so, we sought to compare the journal articles that newspapers covered against a sample of journal articles from highly cited medical journals that could have received media attention.

### Original articles covered by widely circulated newspapers

We analyzed original articles from medical journals that received coverage in the top five newspapers by daily circulation based on the Audit Bureau of Circulations. These newspapers were *The Wall Street Journal*, *USA Today*, *The New York Times*, *Los Angeles Times*, and *San Jose Mercury Times* [9]. From the online version of each newspaper, we prospectively retrieved the first 15 articles in chronological order that covered a clinically-oriented original investigation in a medical journal. Rarely, news stories cover seminal journal articles published years ago; thus, in order to ascertain what new research was being covered, we restricted our analysis to newspaper stories covering a journal article that was published in the preceding 30 days. We defined clinically-oriented research based on the National Institutes of Health definition [10] of “clinical research,” which comprises research with human subjects that is: 1.) patient-oriented research (i.e. investigator interacts directly with human subject); 2.) epidemiological or behavioral research; or 3.) outcomes research and health services research. If several news stories (often from different newspapers) covered the same original investigation, the journal article was only used once in our analysis to ensure that the study sample included 75 unique entries. The corresponding publication cited by each newspaper article was downloaded, and its data were retrieved.

### Original articles in highly cited medical journals

Using *Journal Citation Reports* for the most recent year available (2011), we identified the top five clinical journals by impact factor under the category “General and Internal Medicine”. These journals included *The New England Journal of Medicine*, *The Lancet*, *Journal of the American Medical Association*, *Annals of Internal Medicine*, and *PLoS Medicine*. Within each journal, we prospectively retrieved the first 15 clinically-oriented original publications in chronological order.

### Data extraction

We extracted the following information from each publication: journal, journal impact factor, number of people studied, whether pharmaceutical funding was involved, whether a study was cross-sectional or longitudinal, length of follow-up (if applicable), and whether mortality was examined as an endpoint. We further determined whether each article was an RCT or an observational study (defined as an original study that is not an RCT, meta-analysis, decision or cost-effectiveness analysis, or a study whose main data were derived from modeling - a definition used in prior empirical work) [11]. A five-point study design rating system was also adapted based on the United States Preventive Services Task Force (USPSTF) hierarchy of evidence criteria [12]. The categories were: 1) properly conducted RCT; 2) well-designed controlled trial without randomization; 3) well-designed cohort, case-control, or cross-sectional study; 4) study with multiple time series with or without the intervention, or dramatic results from an uncontrolled study; or 5) descriptive studies or case reports. Because our study used publically available data, institutional review board approval was not necessary.

### Statistical Analysis

Descriptive statistics were displayed for investigations from both high circulation newspapers and high impact medical journals. Subgroup analysis was performed to determine whether differences between investigations from both sources varied by study type (RCT or observational study). Continuous data were displayed as median and interquartile range because data were not normally distributed. Categorical data were displayed as count and percentage. The Wilcoxon-Mann-Whitney test and chi-square test (or Fisher's exact test when appropriate) were used to compare continuous and categorical variables, respectively. A two-sided p-value<0.05 was considered statistically significant. Analysis was performed using Stata v.12 (StataCorp).

## Results

We examined 150 journal articles that either received coverage in widely circulated newspapers (N = 75) or appeared in high impact general medical journals (N = 75) over the same temporal period. Descriptive characteristics of the journal articles based on source (newspaper or high impact journal) are displayed in **Table 1**. Investigations covered by newspapers appeared in journals with a lower impact factor than the group of general internal medicine journals studied (median: 5.4 vs. 30.0, p<0.001). The most common medical journals cited by the media included *New England Journal of Medicine* (16%), *Journal of the American Medical Association* (7%), and *Health Affairs* (5%).

**Table 1:** Characteristics of Clinical Investigations Covered by High Circulation Newspapers and High Impact Medical Journals.

Characteristic	Investigations from High Circulation Newspapers (N = 75)	Investigations from High Impact Medical Journals (N = 75)	P-value
Journal impact factor*	5.4 (4.1–30.0)	30.0 (16.7–33.8)	<0.001
Participants, n*	1034 (112–17408)	1901 (412–32608)	0.14
Pharmaceutical funding, n (%)	5 (7)	12 (16)	0.12
Randomized controlled trials, n (%)	13 (17)	26 (35)	0.016
Observational studies, n (%)	56 (75)	35 (47)	<0.001
Studies assessing mortality, n (%)	17 (23)	19 (25)	0.70
Cross-sectional studies, n (%)	45 (60)	22 (29)	<0.001
Length of follow-up in longitudinal studies, y*	1.80 (0.42–6.00)	1.00 (0.21–4.00)	0.22
Study design rating, n (%)†			0.003
• 1	13 (17)	30 (40)	
• 2	5 (7)	0 (0)	
• 3	51 (68)	38 (51)	
• 4	2 (3)	1 (1)	
• 5	4 (5)	6 (8)	

Presented as median (25^th^–75^th^ percentile) because data was not normally distributed.

Refer to text for details of quality scale.

**Figure 1:**
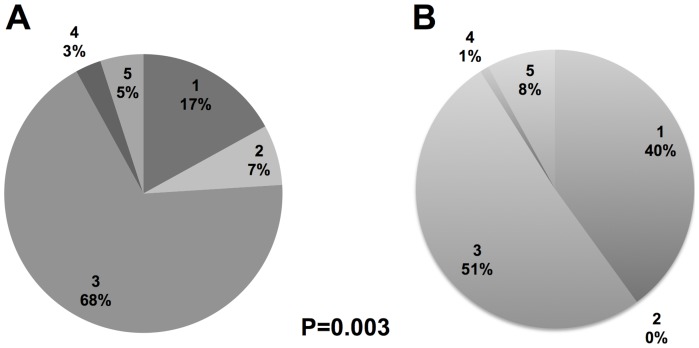
Distribution of study design ratings for clinical investigations from the media and medical journals. (A) The media covers inferior quality study designs than those published in (B) high impact medical journals (p = 0.003; see text for details).

Investigations from the media were less likely to be RCTs (17% vs. 35%, p = 0.016) and more likely to be observational studies (75% vs. 47%, p<0.001) and cross-sectional studies (60% vs. 29%, p<0.001). **Figure 1** depicts the breakdown of study design ratings using the USPSTF hierarchy of evidence for investigations from the media and high impact medical journals, demonstrating that studies from the media were of inferior study design (p = 0.003). No difference was observed in number of subjects studied (median: 1034 vs. 1901, p = 0.14), percent with a pharmaceutical company source of funding (7% vs. 16%, p = 0.12), number of studies assessing mortality (23% vs. 25%, p = 0.70), or length of follow-up (median: 1.80 vs. 1.00 years, p = 0.22) for investigations from the media and medical journals, respectively.

Subgroup analysis stratified by study design (observational study and RCT) is displayed in **Table 2**. Observational studies from the media were published in journals with lower impact factors (median: 5.4 vs. 16.7, p<0.001), smaller sample sizes (median: 1984 vs. 21136, p = 0.029), and were more likely to be cross-sectional (71% vs. 31%, p<0.001), while no differences were observed for RCTs.

**Table 2:** Subgroup Analysis of Observational Studies and Randomized Controlled Trials Stratified by Initial Source of Investigation.

Characteristic	Observational Studies (N = 91)			Randomized Controlled Trials (N = 39)		
	Newspapers (N = 56)	Medical Journals (N = 35)	P-value	Newspapers (N = 13)	Medical Journals (N = 26)	P-value
Journal impact factor*	5.4 (3.9–14.5)	16.7 (16.7–30.0)	<0.001	11.5 (4.7–53.3)	33.8 (30.0–53.3)	0.09
Participants, n*	1984 (173–57491)	21136 (1655–264758)	0.029	420 (84–1020)	568 (312–1723)	0.30
Pharmaceutical funding, n (%)	3 (6)	1 (3)	0.64	2 (15)	10 (38)	0.27
Studies assessing mortality, n (%)	13 (23)	9 (26)	0.79	3 (23)	8 (31)	0.72
Cross-sectional studies, n (%)	40 (71)	11 (31)	<0.001	2 (15)	0 (0)	0.11
Length of follow-up in longitudinal studies, y*	5.50 (2.50–10.13)	3.50 (0.08–10.10)	0.33	0.42 (0.23–1.00)	1.00 (0.25–1.00)	0.36

Presented as median (25^th^–75^th^ percentile) because data was not normally distributed.

## Discussion

Media outlets must make choices when deciding which studies deserve public attention. We sought to examine if there exists a systematic bias favoring certain study design in the choice of articles covered in the press. Our results suggest such a bias; the media is more likely to cover observational studies and less likely to report RCTs than a reference of contemporary articles that appear in high impact journals. When the media does cover observational studies, it selects those with lower sample sizes than observational studies appearing in high impact journals.

While it may not be surprising that the media tends to select articles outside of the highest impact journals, in doing so, they preferentially choose articles lower in the hierarchy of research design, thus favoring studies of lesser scientific credibility. If anything, as top newspapers have their pick of all original articles, not just those selected by high impact general medical journals, newspapers could choose to cover the most credible studies, i.e. large, well-done RCTs. Instead, collectively they appear to make an alternative decision.

Here we present evidence supporting a novel form of selective reporting: [13] the selective reporting of original research articles in the lay press, which emphasizes results from observational studies and minimizes the results of RCTs. Previous work identified a similar percentage of RCTs covered by newspapers (21%), but did not provide a comparison group to determine whether this percentage would be expected based upon the publishing profile of top medical journals [14]. As observational studies have yielded several incorrect conclusions in the history of biomedicine [15], [16], and as RCTs offer the strongest truth claims in all of medicine [17], we provide evidence that the media tends to select articles of weaker methodology.

The media plays an important role in how the average citizen understands their health and emerging health technologies [18]. The majority of Americans follow health news [18], and news coverage translates into real differences in the behavior of Americans. For instance, a surge in local news stories regarding invasive group A streptococcal (GAS) disease, commonly dubbed, “the flesh eating bacteria,” was mirrored by a doubling in GAS testing in a pediatric emergency department, though the number of patients with symptoms warranting testing was unchanged [19]. In addition, a Cochrane review found evidence that the favorability of news coverage was associated with the medical service utilization by providers and patients [20]. Finally, media coverage even affects the influence of research among medical scientists. A seminal study [21] examined articles in the *New England Journal of Medicine*, which were covered in the *New York Times*. A strike by the staff of the *New York Times* in 1978 served as a natural experiment, and over the course of 12 weeks, the newspaper kept a list of articles they intended to cover (but unfortunately could not). The authors found that the *New England Journal of Medicine* articles covered by the *New York* Times received 72.8% more citations than articles that were not covered one year after publication. This effect was not present for articles that the *New York Times* intended to cover, suggesting that coverage encouraged future citations, and not simply that the *New York Times* chose to cover more influential articles.

Others may argue that it is not the role of newspapers to assess the value of medical research at all, but simply to report findings that are of interest to patients and the public. However, there has been renewed focus on improving the media coverage of medical articles in recent years, emphasizing better descriptions of the strengths and weaknesses of medical research. Recent efforts by journalists themselves [22] to strengthen the validity of scientific reporting are in line with these efforts. While discussing strengths and weakness is important, some weaknesses—such as study design—are hard wired. They cannot be adjusted for after the fact, but only acknowledged. Thus, the choice of studies covered by the media is an unexplored way to improve coverage. One potential next step would be utilizing expert consultation from uninvolved researchers for more than providing quotes regarding studies newspapers have decided to cover; they may also provide guidance regarding what studies are worthy of coverage in the first place. The effects of this recommendation however must be tested prospectively before widespread implementation.

The choice of articles covered by the media is the subject of limited research. Previous studies have shown that press releases strongly influence media coverage [23], [24]. In a study of 113 press releases about clinically-oriented publications, 17% promoted meta-analyses or RCTs, while 47% promoted observational studies [25]. Other research shows that improved quality of press releases translates into better quality of subsequent article [26]. Our work suggests that further research is needed to understand the factors that lead institutions and journals to prioritize some studies in press release, but not others.

Of course, newspapers also select stories not just because of their methodological rigor, but also based upon perceptions regarding their potential appeal to readers. For instance, the average reader may be eager to know whether eating a diet heavy in berries lowers cardiovascular risk [27], no matter what methods were employed [11]. For this reason, we compared newspapers not against the gold standard of whether they covered only the best studies that appeared during a given timespan, but a far more achievable bronze standard of whether they covered articles at least as good as studies published in general medical, high impact journals.

There are several limitations to our current study. We examined only a snapshot of stories that appeared in the media, specifically those in widely circulated newspapers. Whether our results apply to other newspapers with smaller circulations or media coverage in other forms of media (television, radio) is unknown. However, as many news outlets select stories based upon the lead of large, highly regarded newspapers [21], we feel that a broader examination of the media is unlikely to yield significantly different results. Also, as we examined only newspaper articles that concerned clinically-oriented health care investigations, and used a hierarchy of experimental design meant for this purpose, our results cannot be generalized to all science coverage.

Additionally, we used the hierarchy of evidence favored by the USPSTF [28] and supported by the Grading of Recommendations Assessment, Development and Evaluation (GRADE) Working Group [29]. While many researchers [30] would not question our choice, it must be acknowledged that some [31] object to this ranking and attribute much more favorable validity to observational studies [32]. It is beyond the scope of our current investigation to recapitulate the long and ongoing debate [30] regarding the reproducibility of observational studies, but note that our position is similar to well respected international groups and advisory bodies.

Our investigation cannot draw conclusions regarding the median impact factor journals that newspapers cover, other than the obvious—they do not exclusively cover those with highest impact. It is entirely reasonable that newspapers would consider articles that appear outside the highest impact journals; however, our results show, that in doing so, they preferentially choose articles of lower sample size and less rigorous study design.

Finally, in our investigation, we used study design (observational, cross-sectional, RCT, etc.) as a surrogate for research quality. This assumption likely only represents a crude assessment of quality. RCTs can be done poorly, yielding incorrect results, while well-conducted observational studies may yield reliable results. Newspapers may have chosen only the finest observational studies, and excluded randomized trials with poor follow up, low sample sizes, and hasty termination. However, within subgroup analysis, our results actually suggested the opposite. Thus, we find evidence for this alternative explanation not compelling. A final, unexplored hypothesis is that, as we did not extract information regarding the prevalence of diseases covered by the media, and the prevalence of diseases covered by high impact journals, the media focuses on articles concerning more prevalent conditions. This is worthy of study in future research.

## Conclusion

In summary, we found that newspapers were more likely to cover observational studies and less likely to cover randomized trials than high impact journals. Additionally, when the media does cover observational studies, they select articles of inferior quality. We present evidence that newspapers preferentially cover medical research with weaker methodology. Our findings add to the understanding of how journalists and medical researchers weight studies. Ultimately such understanding may facilitate communication between researchers and the media and promote coverage that is in the greatest interest of the public health.
